# Neuronal nitric oxide synthase regulation of calcium cycling in ventricular cardiomyocytes is independent of Ca_v_1.2 channel modulation under basal conditions

**DOI:** 10.1007/s00424-019-02335-7

**Published:** 2019-12-10

**Authors:** Janine Ebner, Michal Cagalinec, Helmut Kubista, Hannes Todt, Petra L. Szabo, Attila Kiss, Bruno K. Podesser, Henrietta Cserne Szappanos, Livia C. Hool, Karlheinz Hilber, Xaver Koenig

**Affiliations:** 1 Department of Neurophysiology and-Pharmacology, Center for Physiology and Pharmacology, Medical University of Vienna, Schwarzspanierstraße 17, 1090 Vienna, Austria; 2 Department of Cellular Cardiology, Institute of Experimental Endocrinology, Biomedical Research Center, University Science Park for Biomedicine, Slovak Academy of Sciences, Bratislava, Slovakia; 3 Institute of Molecular Physiology and Genetics, Centre of Biosciences, Slovak Academy of Sciences, Bratislava, Slovakia; 4 Ludwig Boltzmann Cluster for Cardiovascular Research at the Center for Biomedical Research, Medical University of Vienna, Vienna, Austria; 5 School of Human Sciences, The University of Western Australia, Crawley, WA 6009 Australia; 6 Victor Chang Cardiac Research Institute, Darlinghurst, NSW 2010 Australia

**Keywords:** Calcium cycling, Ca_v_1.2 channel regulation, Neuronal nitric oxide synthase, Single-channel recordings, Ventricular cardiomyocytes, Whole cell patch clamp

## Abstract

Neuronal nitric oxide synthase (nNOS) is considered a regulator of Ca_v_1.2 L-type Ca^2+^ channels and downstream Ca^2+^ cycling in the heart. The commonest view is that nitric oxide (NO), generated by nNOS activity in cardiomyocytes, reduces the currents through Ca_v_1.2 channels. This gives rise to a diminished Ca^2+^ release from the sarcoplasmic reticulum, and finally reduced contractility. Here, we report that nNOS inhibitor substances significantly increase intracellular Ca^2+^ transients in ventricular cardiomyocytes derived from adult mouse and rat hearts. This is consistent with an inhibitory effect of nNOS/NO activity on Ca^2+^ cycling and contractility. Whole cell currents through L-type Ca^2+^ channels in rodent myocytes, on the other hand, were not substantially affected by the application of various NOS inhibitors, or application of a NO donor substance. Moreover, the presence of NO donors had no effect on the single-channel open probability of purified human Ca_v_1.2 channel protein reconstituted in artificial liposomes. These results indicate that nNOS/NO activity does not directly modify Ca_v_1.2 channel function. We conclude that—against the currently prevailing view—basal Ca_v_1.2 channel activity in ventricular cardiomyocytes is not substantially regulated by nNOS activity and NO. Hence, nNOS/NO inhibition of Ca^2+^ cycling and contractility occurs independently of direct regulation of Ca_v_1.2 channels by NO.

**Electronic supplementary material:**

The online version of this article (10.1007/s00424-019-02335-7) contains supplementary material, which is available to authorized users.

## Introduction

During the plateau phase of the ventricular action potential, Ca^2+^ influx through Ca_v_1.2 L-type Ca^2+^ channels into the cytosol of cardiomyocytes elicits Ca^2+^-induced Ca^2+^ release from the sarcoplasmic reticulum (SR), which finally triggers contraction. This process is called excitation-contraction (EC) coupling. Various regulatory mechanisms control Ca^2+^ cycling and contractility in ventricular cardiomyocytes. Among those, enhancement of the currents through Ca_v_1.2 channels by the sympathetic nervous system via activation of β-adrenergic receptors during the so-called fight-or-flight response [7, 9, 11] is the most prominent and best established.

Besides upregulation of Ca_v_1.2 activity in response to β-adrenergic signalling, neuronal nitric oxide synthase (nNOS) is also considered a regulator of Ca_v_1.2 and downstream Ca^2+^ cycling in the heart. The most widely accepted view is that nitric oxide (NO), generated by nNOS activity in cardiomyocytes, reduces the currents through Ca_v_1.2 channels (e.g. [5, 25, 26, 33]). This gives rise to a diminished Ca^2+^ release from the SR, as reflected by a smaller amplitude of intracellular Ca^2+^ transients [5, 19, 25], and finally reduced contractility [6, 25, 26]. Evidence for Ca_v_1.2 inhibition by nNOS activity has been derived from studies using nNOS−/− mice and/or pharmacological inhibitors of the enzyme. Ventricular cardiomyocytes derived from nNOS−/− mice showed significantly bigger L-type Ca^2+^ currents than myocytes from control mouse ventricles [5, 25, 31]. In accordance, pharmacological inhibition of nNOS activity in rodent ventricular cardiomyocytes increased Ca^2+^ current amplitudes [5, 12, 25, 32], and the application of NO donors reduced Ca_v_1.2 currents [5, 13]. Collectively, these studies suggested that NO, generated by nNOS activity, reduces the currents through Ca_v_1.2 channels in the heart, and this effect was linked with S-nitrosylation of the channel protein [5, 28].

In recent years, we have attempted to reproduce evidence for nNOS- or NO-mediated regulation of Ca_v_1.2 function in mouse ventricular cardiomyocytes, but—much to our surprise—we have failed. A more thorough literature search then revealed evidence from other groups contradicting the established concept of Ca_v_1.2 inhibition by NO via nNOS activity. For example, Barouch and colleagues [2] reported that cardiomyocytes from nNOS−/− mice exhibit normal Ca^2+^ currents, and the application of NO donors did not significantly affect the Ca^2+^ currents in rat ventricular cardiomyocytes [1] and Ca_v_1.2 channels expressed in HEK293 cells [34]. The apparent inconsistency in the literature concerning nNOS/NO regulation of Ca_v_1.2 channels prompted us to reinvestigate this important issue by using a combination of different methodological approaches.

We find that nNOS/NO activity alters intracellular Ca^2+^ transients but not through a direct effect on Ca_v_1.2 channels. We conclude from our results that—against the currently prevailing view—basal Ca_v_1.2 channel activity in ventricular cardiomyocytes is not substantially regulated by nNOS activity and NO. Hence, nNOS/NO regulation of Ca^2+^ cycling and contractility in myocytes can occur independent of direct effects of NO on Ca_v_1.2 channel function.

## Materials and methods

### Isolation of ventricular cardiomyocytes

Male C57BL/10ScSnJ mice (15–25 weeks of age) and female Sprague Dawley rats (12–14-week-old) were killed by cervical dislocation. Cardiomyocytes were isolated from the ventricles of their hearts using a Langendorff setup as described in our previous article [15], and plated on Matrigel (Becton Dickinson)-coated culture dishes. Some myocytes of each preparation were immediately used for intracellular Ca^2+^ transient measurements, and some for patch clamp recordings (see below). Myocytes from at least 3 preparations (or animals) were used for each individual experiment performed.

### Intracellular Ca^2+^ transient measurements

Intracellular Ca^2+^ transients were recorded from electrically stimulated ventricular cardiomyocytes, up to 8 h after preparation, using the Ca^2+^-sensitive fluorescent dye fluo-4 AM (Thermo Fisher Scientific). The respective experimental and analysis procedures are described in our recently published article [24]. The myocytes were bathed in an extracellular solution containing (in mmol/L) 140 NaCl, 4 KCl, 2 CaCl_2_, 2 MgCl_2_, 5 HEPES, 5 Glucose and pH adjusted to 7.4 with NaOH.

### Ca^2+^ and Ba^2+^ current recordings

Currents were recorded in the conventional whole cell or in the perforated patch mode of the patch clamp technique from cardiomyocytes up to 8 h after preparation, at an experimental temperature of 22 ± 1.5 °C, using an Axoclamp 200B patch clamp amplifier (Axon Instruments). Pipettes were pulled from aluminosilicate glass capillaries (A120-77-10; Science Products) with a P-97 horizontal puller (Sutter Instruments), and had resistances between 1.2 and 2 MΩ when filled with pipette solution (see below). Data acquisition was performed with the pClamp 10 software (Axon Instruments) through a 16-bit A-D/D-A interface (Digidata 1440; Axon Instruments). Data were low-pass filtered with 2 kHz (3 dB) and digitized at 5 kHz. Leak currents and capacity transients were subtracted using a P/4 protocol. Data analyses were executed with Clampfit 10.2 (Axon Instruments) and GraphPad Prism 5.01 software. The external (bath) solution for Ca^2+^ current measurements contained (in mmol/L) 2 CaCl_2_, 157 TEA-Cl, 10 HEPES and pH 7.4 adjusted with TEA-OH. The bath solution for Ba^2+^ current measurements contained 10 BaCl_2_, 145 TEA-Cl, 10 HEPES and pH 7.4 adjusted with TEA-OH. A series of control experiments was performed with a bath solution containing 140 NaCl, 4 KCl, 2 CaCl_2_, 2 MgCl_2_, 5 HEPES, 5 Glucose and pH adjusted to 7.4 with NaOH. The internal (pipette) solution consisted of 145 Cs-aspartate, 2 MgCl_2_, 10 HEPES, 0.1 Cs-EGTA, 2 Mg-ATP and pH 7.4 adjusted with CsOH. For perforated patch experiments, pipettes were front-filled with internal solution and then back-filled with the same solution containing 500 μg/ml amphotericin B. After establishment of a GΩ seal, the series resistance was monitored until it stabilized below 20 MΩ. This typically happened after 10–20 min. Ca^2+^ and Ba^2+^ currents were elicited from a holding potential of − 80 mV by depolarizing voltage steps up to + 50 mV. For the determination of current density–voltage relationships, the current amplitudes at various voltages were measured. These were divided by the cell capacitance to obtain current densities, which were plotted against the respective test pulse voltages. The kinetics of Ca^2+^ current inactivation (representing both Ca^2+^- and voltage-dependent channel inactivation) was analysed by measuring the time period between the current peak and the time point at which the current had decayed to 50%. This parameter, also used in our previous articles [16, 24], is termed “decay half-time”. Ba^2+^ current inactivation (representing only voltage-dependent Ca^2+^ channel inactivation) was analysed as follows: the current decay after channel activation was fit with a single exponential function to derive respective time constants (tau-values). Ba^2+^ current inactivation kinetics were only derived from the experiments described in Fig. 4, because the short duration of the test pulses (50 ms) applied in the other sets of experiments (Fig. 3, Table 1) precluded a valid determination of tau-values. Current–voltage relationships were fit with the function: *I*(*V*) = *G*_max_ ·(*V* − *V*_rev_)/{1 + exp.[(*V*_0.5_ − *V*)/*K*]}, where *I* is the current, *G*_max_ is the maximum conductance, *V* is the membrane potential, *V*_rev_ is the reversal potential, *V*_0.5_ is the voltage at which half-maximum activation occurred, and *K* is the slope factor.

**Table 1:** External application of NOS inhibitors and the NO donor SNAP does not affect whole cell currents through L-type Ca2+ channels in mouse ventricular cardiomyocytes. The rundown-corrected current amplitudes are expressed in percentage relative to the respective value at experiment start (= 100%). Decay half-time represents the time period between the current peak and the time point at which the current had decayed to 50%. Values are expressed as means ± SD, and the number of experiments performed (*n*) is given in brackets. The control (ctl) values were detected immediately before drug application, and the experimental values (drug) were taken 180 s after the start of superfusion with the respective drug. The “wash” values were detected 180 s after the start of drug washout. Ca^2+^ and Ba^2+^ indicate the use of bath solution containing 2 mM Ca^2+^ or 10 mM Ba^2+^, respectively. The respective concentrations of the drugs used are given in brackets in the leftmost column. No significant difference existed between ctl and drug-treated cells (*p* values always > 0.2, paired Student’s *t* test). The only exception (*p = 0.01, ctl versus NPLA Ca^2+^ current decay half-time) is indicated with an asterisk. For current amplitude value comparisons, *t* tests were performed on the raw data before normalization

	Current amplitude (%, rel. to value at experiment start)			Current decay (decay half-time, ms)		
	ctl	drug	wash	ctl	drug	wash
NPLA (1 mM)						
Ca^2+^	100 ± 8 (6)	105 ± 12 (6)	105 ± 11 (5)	8,3 ± 0,9 (6)	9,3 ± 1,0 (6)*	9,1 ± 1,5 (5)
Ba^2+^	96 ± 4 (5)	98 ± 5 (5)	101 ± 4 (5)			
L-NMMA (1 mM)						
Ca^2+^	93 ± 7 (7)	95 ± 9 (7)	97 ± 5 (7)	9,9 ± 2,0 (7)	9,6 ± 2,0 (7)	9,7 ± 1,9 (7)
Ba^2+^	102 ± 4 (10)	101 ± 4 (10)	100 ± 5 (9)			
SNAP (10 μM)						
Ca^2+^	101 ± 18 (8)	106 ± 27 (8)	104 ± 27 (8)	9,3 ± 1,9 (8)	9,6 ± 1,8 (8)	9,8 ± 1,7 (8)
SNAP (500 μM)						
Ca^2+^	108 ± 43 (7)	111 ± 49 (7)	105 ± 19 (5)	9,8 ± 2,0 (7)	9,9 ± 1,8 (7)	8,8 ± 3,2 (5)

### Rundown correction procedure

Both Ca^2+^ and Ba^2+^ currents in cardiomyocytes, detected with the conventional whole cell patch clamp technique, regularly showed considerable rundown, when recorded over prolonged time periods (up to several min). To correct for this rundown, in each experiment, a single exponential function was fit through all data points under control conditions (for examples see Fig. 2c and d, left). This function was then used to time-dependently correct every actual-measured current amplitude peak value over the whole recording period. This typically resulted in constant current amplitudes until the end of a recording (Fig. 2c and d, right). Only if rundown correction provided a satisfactory result comparable with the experiments shown in Fig. 2c and d, drug superfusion experiments were used for analyses of drug effects. Rundown correction was not necessary in the experiments performed with the perforated patch clamp technique (Fig. 5). In Figs. 3 and 5, as well as in Table 1, relative current amplitude values are given in percentage with respect to the value at experiment start (100%).

### Single-channel patch clamp on proteoliposomes

For the proteoliposome experiments, the human cardiac L-type voltage-gated Ca^2+^ channel α1 subunit long N-terminal (CAC1C_HUMAN isoform 34, Q13936–34, long-NT) variant was used. A pGEM-HJ vector containing the cDNA of the long NT isoform of the α1C subunit of Ca_v_1.2 (α1C,77L) was a gift from N. Dascal (Tel Aviv University, Israel) and N. Soldatov (National Institutes of Health, Baltimore, MD, USA). The cDNA of the long N-terminal isoform of the Ca_v_1.2 was cloned into pcDNA3.1 vector (Invitrogen) and modified to include a HIS6 tag at the N-terminus. More details are available in [9, 20, 30]. The 6X His-tagged protein was expressed in HEK293T cells, then purified by Ni-NTA Agarose beads. Purified proteins were incorporated in artificial liposomes in 1:1000 protein/lipid ratio, following the dehydration/rehydration method as previously described [9]. Single-channel mode of patch clamp technique was used to record openings of the channel. Recording and pipette solutions contained 50 mM NaCl, 100 mM BaCl_2_, 10 mM HEPES and 2 μM BayK8644 (pH = 7.4). The back-filled microelectrode had an average resistance of 16–17 MΩ. Single-channel currents were filtered at 1 kHz, digitized at 100 kHz and analysed using pClamp software (Molecular Devices). The Ca_v_1.2 channel was determined by the magnitude of the current, changes in open probability of the channel (Po) and sensitivity of the current to the L-type Ca^2+^ channel antagonist nisoldipine. Current traces from control condition and after 150 μM GSNO (S-nitrosoglutathione) or 100 μM SNP (sodium nitroprusside) as NO donor treatment, or 0.5 μM protein kinase A (PKA, catalytic subunit, reduced 1 mM DTT, 5 mM NEM, substituted 1 mM ATP-disodium salt) were compared relative to control solution within the same patch.

### Drug sources and application

#### NOS inhibitors

**Fig. 1:**
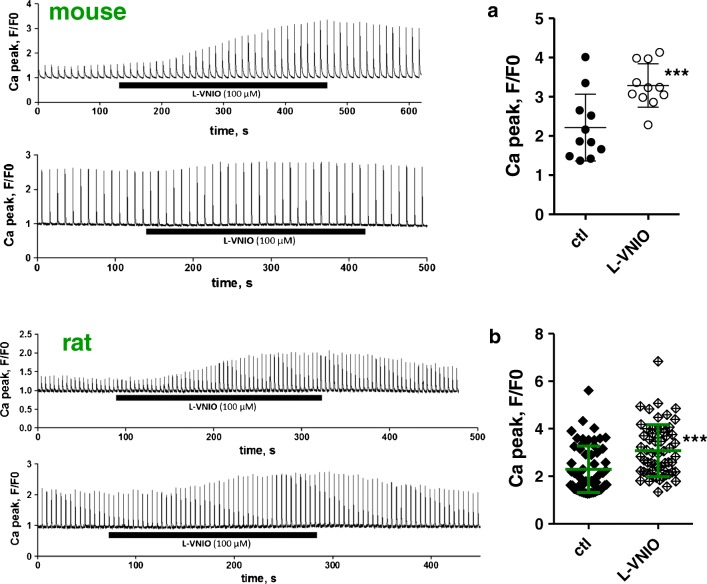
External application of the nNOS inhibitor L-VNIO increases electrically induced cytosolic Ca^2+^ transients in mouse (**a**) and rat (**b**) ventricular cardiomyocytes. Left: representative time courses of Fluo-4 fluorescence reporting rises in cytosolic Ca^2+^ concentration during electrical field stimulation, at 0.1 and 0.2 Hz frequency, in single mouse (**a**) and rat (**b**) ventricular cardiomyocytes, respectively. Following a time period under control conditions, the cells were superfused with bath solution containing 100 μM L-VNIO for the respective indicated durations (see black bars). Right: mean Ca^2+^ peak fluorescence relative to baseline (F/F0) elicited by electrical field stimulation for mouse (**a**) and rat (**b**) myocytes in the absence (ctl) and the presence (L-VNIO) of 100 μM of the drug. Each data point represents a single cell (*n* = 11 and 59 for mouse and rat cardiomyocytes, respectively), and values are expressed as means (calculated from these total cell numbers) ± SD. Cells originated from four individual mouse and nine rat hearts in total. Triple asterisks indicate a significant difference between the drug-free control condition and the presence of L-VNIO (*p* < 0.001, paired Student’s *t* test)

In accordance with previously published studies, cell permeable Vinyl-L-NIO (hydrochloride) (L-VNIO; Cayman Chemical, CAY-80330-5) (e.g. [5, 6, 25]) and cell permeable N (ω)-propyl-l-arginine (NPLA; Abcam, ab146405) [19, 22] were used as selective nNOS inhibitors. Cell permeable NG-monomethyl-l-arginine (monoacetate salt) (L-NMMA; Abcam, ab120137) was used as unselective NOS inhibitor as in [12, 18]. L-VNIO was dissolved in DMSO; the drug-free control bath solutions contained the same amount of DMSO as the experimental solutions. NPLA and L-NMMA were dissolved in H_2_O. In order to allow the drugs to sufficiently penetrate to the cytoplasm, external superfusion of cardiomyocytes with NOS inhibitors was always performed for at least 180 s. This treatment duration was considered sufficient because, in the intracellular Ca^2+^ transient measurements, maximum NOS inhibitor effects typically occurred not later than 180 s after the start of superfusion with drug (see Fig. 1).

#### NO donors

S-Nitroso-N-acetyl-DL-penicillamine (SNAP; Sigma, n3398) was used as NO donor in the cardiomyocyte experiments. SNAP was solved in DMSO; the drug-free control bath solutions contained the same amount of DMSO as the experimental solutions. Another S-nitrosothiol compound the S-nitroso-l-glutathione (GSNO, Cayman Chemical, 82,240) was used in the single-channel experiments [27]. Stock solution was made by using DMSO. Since many studies have suggested important role for cell enzymes in GSNO metabolism/NO liberation (γ-glutamyltranspeptidase, superoxide dismutase, glutathione peroxidase, reviewed in [4]) for our cell-free experimental system, sodium nitroprusside dihydrate (SNP, Sigma 71,778) was used as NO donor.

##### Statistics

Data are expressed as means ± SD. Statistical comparisons between drug-free control and drug-treated conditions were made using paired or unpaired (as appropriate, see Figure legends) two-tailed Student’s *t* tests. A *p* < 0.05 was considered significant.

## Results

### Inhibition of nNOS activity increases intracellular Ca^2+^ transients in ventricular cardiomyocytes

nNOS-derived NO has been shown to regulate Ca^2+^ cycling and contractility in ventricular cardiomyocytes. NO decreases Ca^2+^ transients and attenuates contraction (see the “Introduction” section). Here, we first tried to reproduce this well-established nNOS/NO effect in our experimental system. Therefore, we recorded intracellular Ca^2+^ transients in single ventricular cardiomyocytes and studied the effects of cell-permeable nNOS inhibitors. Figure 1 shows that superfusion with the nNOS inhibitor L-VNIO in a concentration of 100 μM significantly increased the amplitude of Ca^2+^ transients recorded from mouse ventricular cardiomyocytes (Fig. 1a). A similar effect was obtained by application of 1 mM NPLA, another cell permeable nNOS inhibitor (data not shown). In addition, we performed experiments with ventricular cardiomyocytes derived from rat hearts. As in the mouse myocytes, nNOS inhibition by L-VNIO increased the amplitude of Ca^2+^ transients in the rat cells (Fig. 1b). Together, these results are consistent with the established inhibitory effect of nNOS/NO activity on Ca^2+^ cycling in ventricular cardiomyocytes.

### nNOS/NO activity does not affect currents through L-type Ca^2+^ channels in ventricular cardiomyocytes

**Fig. 2:**
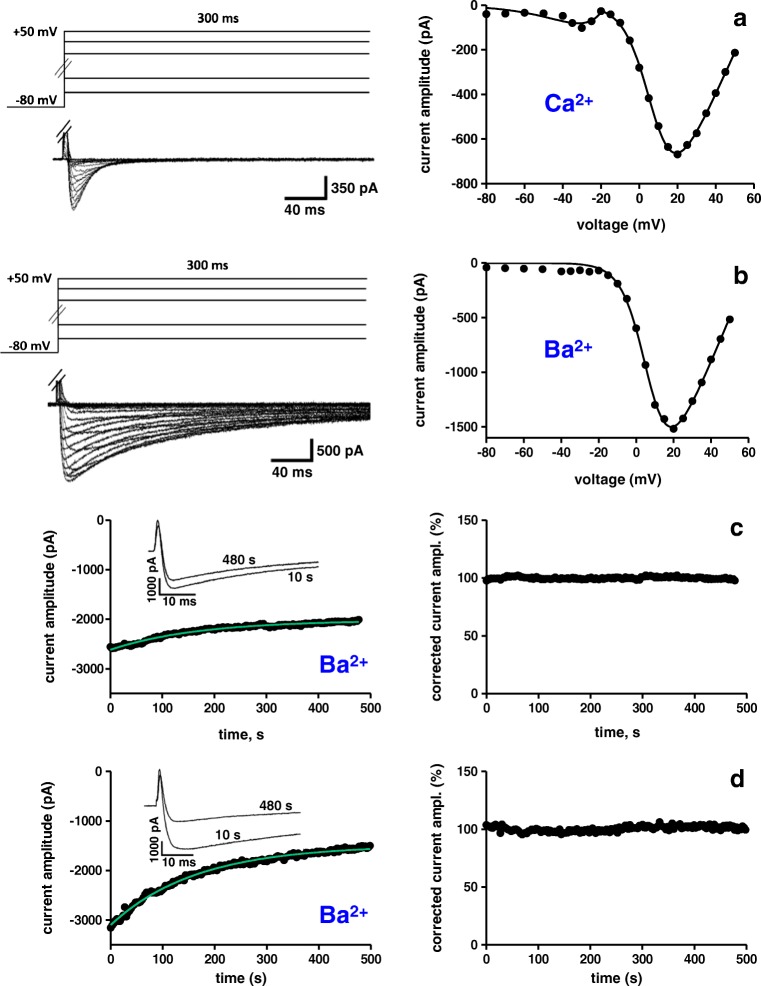
Whole cell currents through Ca^2+^ channels recorded from mouse ventricular cardiomyocytes. **a** The left figure part shows typical original traces of Ca^2+^ currents elicited by the pulse protocol displayed on top. On the right, the respective inward current peaks were plotted against the applied membrane potentials to obtain the current–voltage relationship. The solid line represents a fit of the data points with a function described in the “Materials and methods” section. **b** Original Ba^2+^ current traces (left) and the respective current–voltage relationship (right). **c**, **d** Left: examples of continuous Ba^2+^ current recordings, in the conventional whole cell patch clamp mode, showing permanent rundown over 500 s. The currents were elicited by 50-ms pulses to + 20 mV every 3 s from a holding potential of − 80 mV, and their peaks were plotted against time. The insets display the respective currents at the beginning (time point, 10 s) and the end (time point, 480 s) of the experiments. The solid lines (green) represent fits of the data points with a single exponential function. Right: rundown-corrected current peaks, expressed in percentage relative to the respective value at experiment start (= 100%), were plotted over time. In this control series of experiments, all recorded Ca^2+^ and Ba^2+^ currents (from six individual cardiomyocytes each) showed a similar single exponential rundown over time as the two displayed examples. The rundown correction procedure is described in detail in the “Materials and methods” section

Having proven that nNOS/NO activity exerts the expected effect on Ca^2+^ cycling in our experimental system, we proceeded to study the impact of nNOS/NO on L-type Ca^2+^ channels. Figure 2 shows typical original traces of Ca^2+^ (Fig. 2a, left) and Ba^2+^ (Fig. 2b, left) currents recorded from mouse ventricular cardiomyocytes using the conventional whole cell patch clamp technique. When Ca^2+^ was used as charge carrier, the current–voltage relationships often revealed two distinct peaks: a tiny peak at approximately − 30 mV and a larger peak with a maximum between + 10 and + 20 mV (Fig. 2a, right). These represent T-type and L-type Ca^2+^ channel activity, respectively. Because of the minor amplitude of the occurring T-type Ca^2+^ currents (practically absent in the Ba^2+^ current recordings, Fig. 2b), these were not further considered in the analyses of the experiments described below. When whole cell Ca^2+^ and Ba^2+^ currents, elicited by a voltage step from − 80 to + 20 mV, were recorded over prolonged time periods (up to several min), considerable current rundown was regularly observed. Two examples of Ba^2+^ current rundown are shown in the left part of Fig. 2 c (moderate rundown) and d (strong rundown). In order to enable us to analyse effects of drugs during cardiomyocyte superfusion (and subsequent washout) over prolonged periods of time, we introduced a rundown correction procedure (for description of the applied procedure see the “Materials and methods” section). Figure 2c and d show exemplary analyses of current amplitude over time without (left) and after (right) rundown correction. It can be observed that the applied correction procedure adequately restored the original current amplitude at the beginning of the experiment throughout the whole recording duration. Only if rundown correction provided a satisfactory result comparable with the experiments shown in Fig. 2c and d, drug superfusion experiments (see below) were used for analyses of drug effects.

**Fig. 3:**
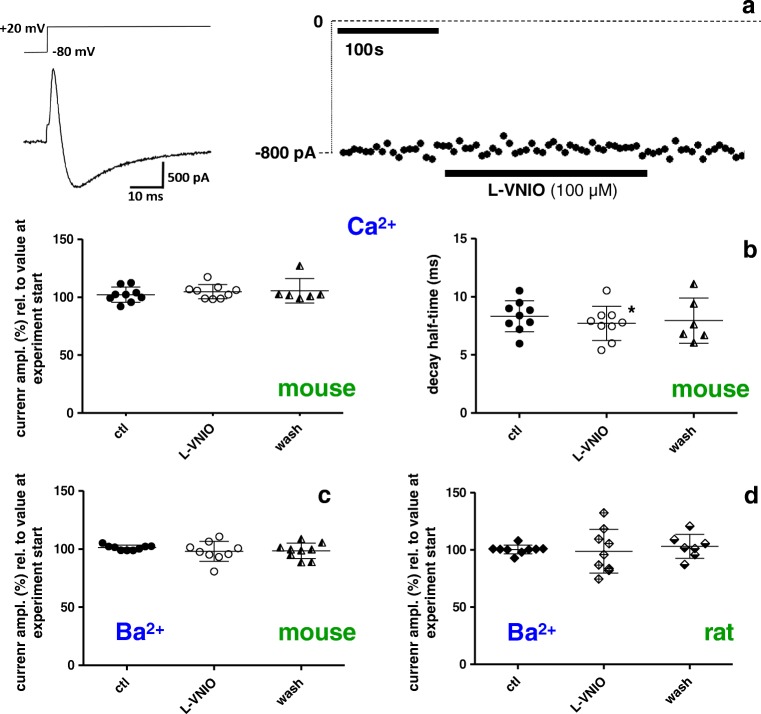
External application of the nNOS inhibitor L-VNIO does not affect currents through L-type Ca^2+^ channels in ventricular cardiomyocytes. **a** Left: original trace of a Ca^2+^ current, elicited by the pulse protocol displayed on top, of a mouse ventricular cardiomyocyte under control (drug-free) condition. Right: the rundown-corrected peaks of the currents, elicited by pulses every 3 s, before drug application, during superfusion with 100 μM L-VNIO, and after drug washout were plotted over time. **b** Evaluation summary of a series of experiments with mouse cardiomyocytes as described in a. Ca^2+^ current amplitudes (left) and decay kinetics (right) in the absence (ctl, wash) and presence of L-VNIO are compared. The control (ctl) amplitude values were detected immediately before drug application, and the experimental values (in the presence of the drug) were taken 180 s after the start of superfusion with L-VNIO. The “wash” amplitude values were detected 180 s after the start of drug washout. The current amplitudes are expressed in percentage relative to the respective value at experiment start (= 100%). Each data point represents a single cell, and data variation is expressed as SD. Decay half-time represents the time period between the current peak and the time point at which the current had decayed to 50%. Drug application did not significantly alter current amplitude (*p* = 0.97, paired Student’s *t* test performed on raw data before normalization; *n* = 9 for ctl, 9 for drug-treated, and 6 for wash). Cells originated from four mouse hearts. A significant difference (*p = 0.02) existed between the decay half-times of ctl and L-VNIO-treated cells. **c** Comparison of Ba^2+^ current amplitudes in mouse cardiomyocytes in the absence and presence of L-VNIO. ctl and drug-treated cells showed no significant difference (*p* = 0.33, paired Student’s *t* test; *n* = 9 for ctl, 9 for drug-treated, and 9 for wash). Cells originated from four mouse hearts. **d** Comparison of Ba^2+^ current amplitudes in rat ventricular cardiomyocytes in the absence and presence of L-VNIO. ctl and drug-treated cells showed no significant difference (*p* = 0.90, paired Student’s *t* test; *n* = 9 for ctl, 9 for drug-treated, and 7 for wash). Cells originated from four rat hearts

In order to test whether nNOS/NO activity impacts basal whole cell currents through L-type Ca^2+^ channels in ventricular cardiomyocytes, we first applied the cell-permeable nNOS inhibitor L-VNIO (Fig. 3). Superfusion of mouse ventricular cardiomyocytes with 100 μM L-VNIO had no significant effect on the current amplitude, independent of the use of either Ca^2+^ (Fig. 3a and b, left) or Ba^2+^ (Fig. 3c) as charge carrier. Ca^2+^ current decay, representing channel inactivation kinetics, was slightly but significantly (*p* = 0.02, paired Student’s *t* test) speeded in the presence of L-VNIO (Fig. 3b, right). We then tested the effects of L-VNIO on rat ventricular cardiomyocytes. Here, we could only manage to study drug effects on Ba^2+^ currents, because the Ca^2+^ current recordings on rat myocytes were too unstable. Consistent with the findings in the mouse, L-VNIO superfusion did not alter the Ba^2+^ current amplitudes in rat myocytes (Fig. 3d).

Next, we tested the effect of another cell-permeable nNOS inhibitor, NPLA. Superfusion of mouse ventricular cardiomyocytes with 1 mM NPLA did not affect the current amplitude (Table 1). As observed with L-VNIO (see above), this was independent of the use of Ca^2+^ or Ba^2+^ as charge carrier. Ca^2+^ current decay was slightly but significantly (*p* = 0.01, paired Student’s *t* test) slowed by NPLA (Table 1).

To test whether the lack of considerable effects of L-VNIO and NPLA on the Ca^2+^ channel properties in ventricular cardiomyocytes was due to the fact that these compounds are selective nNOS inhibitors, we also applied the nonselective cell-permeable NOS inhibitor L-NMMA. Similar to the nNOS inhibitors, superfusion with 1 mM L-NMMA did not affect the Ca^2+^ channel properties in mouse ventricular cardiomyocytes (Table 1). Together, these results suggest that external application of cell-permeable nNOS and/or NOS inhibitors to ventricular cardiomyocytes does not considerably affect their basal L-type Ca^2+^ channel properties.

**Fig. 4:**
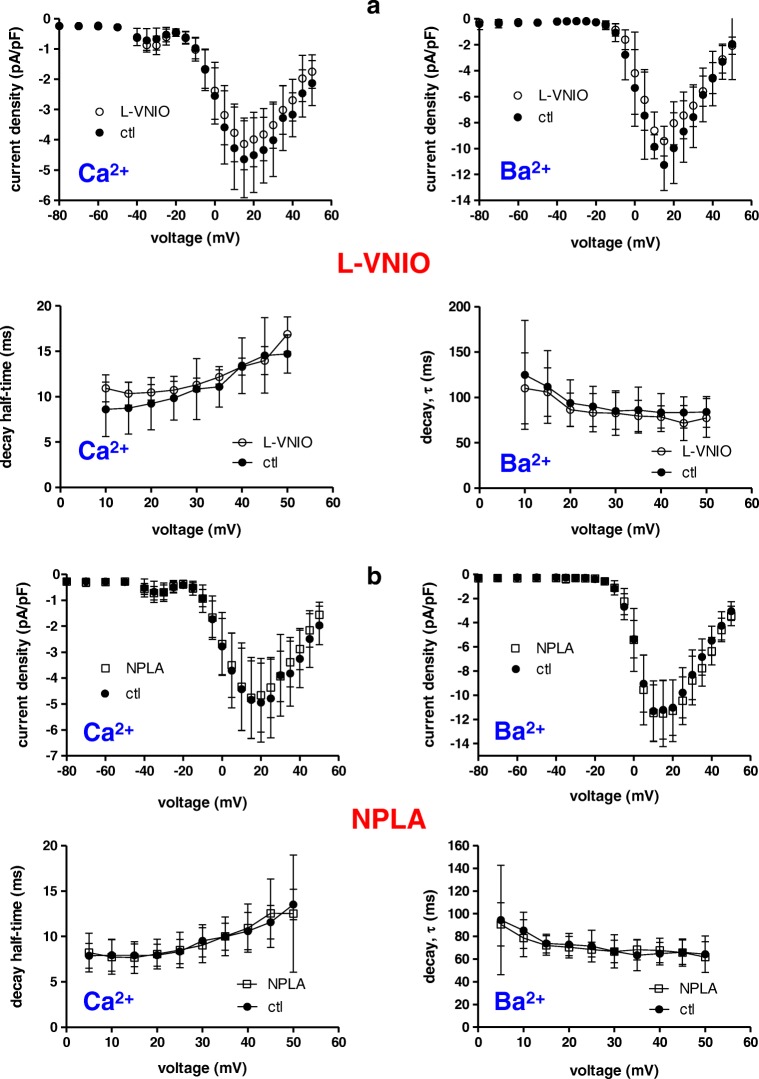
Internal application of nNOS inhibitors via the pipette solution does not affect currents through L-type Ca^2+^ channels in mouse ventricular cardiomyocytes. **a** Left: Ca^2+^ current density–voltage relationships (top) and decay kinetics (bottom) of myocytes recorded under control conditions (standard pipette solution, ctl), or in the presence of 100 μM L-VNIO in the pipette. The currents were elicited by 400-ms pulses to various depolarizing potentials from a holding potential of − 80 mV. Data points are given as means ± SD (*n* = 7 for ctl and 6 for drug-treated cells). Cells originated from three mouse hearts. No significant difference (*p* always > 0.23, unpaired Student’s *t* test) existed between ctl and drug-treated cells over the whole voltage range studied. Right: Ba^2+^ current density–voltage relationships (top) and decay kinetics (bottom; given as time constant tau derived from a single exponential fit of the current decay) of myocytes recorded under control conditions, or in the presence of L-VNIO in the pipette. There was no significant difference (*p* always > 0.10, unpaired Student’s *t* test) between ctl and drug-treated cells at all voltages (*n* = 6 for ctl and 5 for drug-treated). Cells originated from three mouse hearts. **b** Left: Ca^2+^ current density–voltage relationships (top) and decay kinetics (bottom) of cardiomyocytes recorded under control conditions, or in the presence of 100 μM NPLA in the pipette. No significant difference (*p* always > 0.47, unpaired Student’s *t* test) existed between ctl and drug-treated cells over the whole voltage range studied (*n* = 9 for ctl and 12 for drug-treated). Cells originated from three mouse hearts. Right: Ba^2+^ current density–voltage relationships (top) and decay kinetics (bottom) of myocytes recorded under control conditions or in the presence of NPLA in the pipette. There was no significant difference (*p* always > 0.67, unpaired Student’s *t* test) between ctl and drug-treated cells (*n* = 9 for ctl and 9 for drug-treated). Cells originated from three mouse hearts

In order to exclude the possibility that the applied nNOS inhibitor compounds—although cell permeable per se—did not reach the cytoplasm of the myocytes in sufficient concentration, and therefore not modulated L-type Ca^2+^ channel properties, we performed additional experiments where the inhibitors were applied directly to the cytoplasm via the pipette solution. Figure 4a shows Ca^2+^ (top left) and Ba^2+^ (top right) current density–voltage relationships recorded from mouse ventricular cardiomyocytes under control conditions (ctl, standard pipette solution) compared with respective relationships in the presence of 100 μM of the nNOS inhibitor L-VNIO in the pipette. To guarantee sufficient diffusion of the drug into the cytosol via the pipette tip, current–voltage relationships were always only recorded 5 min after whole cell access had been established. The same procedure was also used for experiments in the absence of L-VNIO in the pipette. It can be observed that the current densities were similar in the presence and absence of the drug over the whole range of voltages studied (Fig. 4a, top). Moreover, the Ca^2+^ (Fig. 4a, bottom left) and Ba^2+^ (Fig. 4a, bottom right) current decay kinetics were also independent of the presence of L-VNIO. Besides L-VNIO, we also used the nNOS inhibitor NPLA for an identically performed set of experiments. Figure 4b shows that, similar to L-VNIO, the presence of 100 μM NPLA in the pipette solution did not cause any changes in Ca^2+^ channel properties when compared with the drug-free condition. In summary, our experiments with nNOS/NOS inhibitors revealed no substantial drug effects on basal currents through L-type Ca^2+^ channels in ventricular cardiomyocytes.

In rat ventricular myocytes, NOS inhibition did not affect the basal Ca^2+^ current, but in a cAMP-stimulated condition (due to application of the adenylyl cyclase activator forskolin or the phosphodiesterase inhibitor milrinone), the current was significantly augmented by the application of a NOS inhibitor [18]. Here, using a similar experimental strategy, we tested if external L-VNIO (100 μM) application affected the Ca^2+^ current amplitude in mouse ventricular cardiomyocytes pre-treated with forskolin (5 μM) and milrinone (10 μM) (Suppl. Fig. 1). We found that also in a cAMP-stimulated condition, L-VNIO had no effect on the current amplitude.

As nNOS/NOS inhibition failed to affect L-type Ca^2+^ channels, we reasoned that direct exposure of cardiomyocytes to NO should be ineffective as well. To test this, we applied the NO donor SNAP in two different concentrations to mouse ventricular cardiomyocytes. Neither superfusion with 10 nor 500 μM SNAP generated any significant effects on Ca^2+^ current amplitude or current decay kinetics (Table 1). Together with the nNOS/NOS inhibitor studies, these experiments strongly suggest that nNOS/NO activity does not substantially modulate L-type Ca^2+^ channel properties in ventricular cardiomyocytes.

**Fig. 5:**
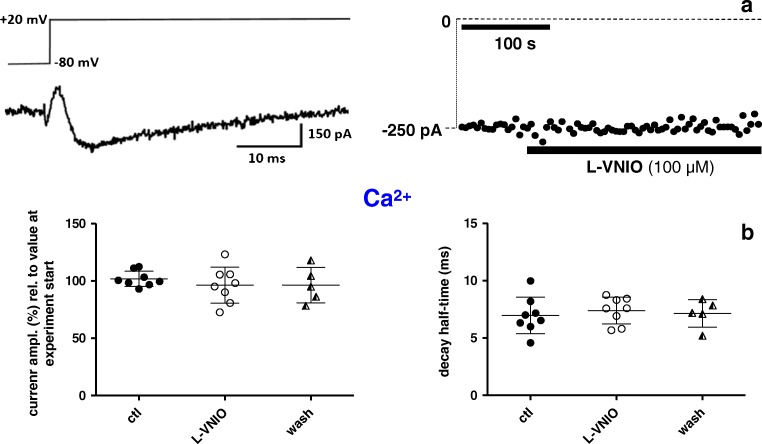
External application of the nNOS inhibitor L-VNIO does not affect Ca^2+^ currents in mouse ventricular cardiomyocytes recorded with the perforated patch clamp technique. **a** Left: voltage-clamp protocol and corresponding original Ca^2+^ current trace under control conditions. Right: maximal inward current amplitude before drug application and during superfusion with 100 μM L-VNIO was plotted over time. **b** Evaluation summary of a series of experiments with mouse cardiomyocytes as described in **a**. Ca^2+^ current amplitudes (left) and decay kinetics (right) in the absence (ctl, wash) and presence of L-VNIO are compared. Drug application did not generate any significant difference (*p* always > 0.36, paired Student’s *t* test; *n* = 8 for ctl, 8 for drug-treated, and 5 for wash). Cells originated from four mouse hearts

In order to guarantee for a proper voltage control over the large and strongly invaginated membrane of adult ventricular cardiomyocytes, all the Ca^2+^ channel current recordings described above were carried out with the conventional whole cell patch clamp technique. A disadvantage of using this technique is that cytoplasmic components, e.g. potentially important signalling molecules, can be gradually lost during the recordings by back diffusion through the pipette tip into the bulk pipette solution. Thus, it is possible that indirectly exerted nNOS/NO effects on the L-type Ca^2+^ channel, i.e. via signalling cascades, were overlooked in the whole cell patch clamp mode. For this reason, we decided to perform control experiments in the perforated patch configuration, which is thought to largely avoid signalling molecule loss through the pipette tip. In contrast to the conventional whole cell recordings, Ca^2+^ currents recorded in the perforated patch clamp mode did not show any rundown over several minutes (for example see Fig. 5a, right). Therefore, it was not necessary to apply a rundown correction procedure before analysing this set of experiments. Consistent with the conventional whole cell patch clamp experiments, in our perforated patch clamp studies, superfusion of mouse ventricular cardiomyocytes with the nNOS inhibitor L-VNIO did not alter their Ca^2+^ current properties (Fig. 5). This result suggests that, also in the presence of intact cytosolic signalling pathways, nNOS/NO activity does not alter the properties of L-type Ca^2+^ channels.

### Human Ca_v_1.2 single-channel currents in artificial membranes are not modulated by NO

**Fig. 6:**
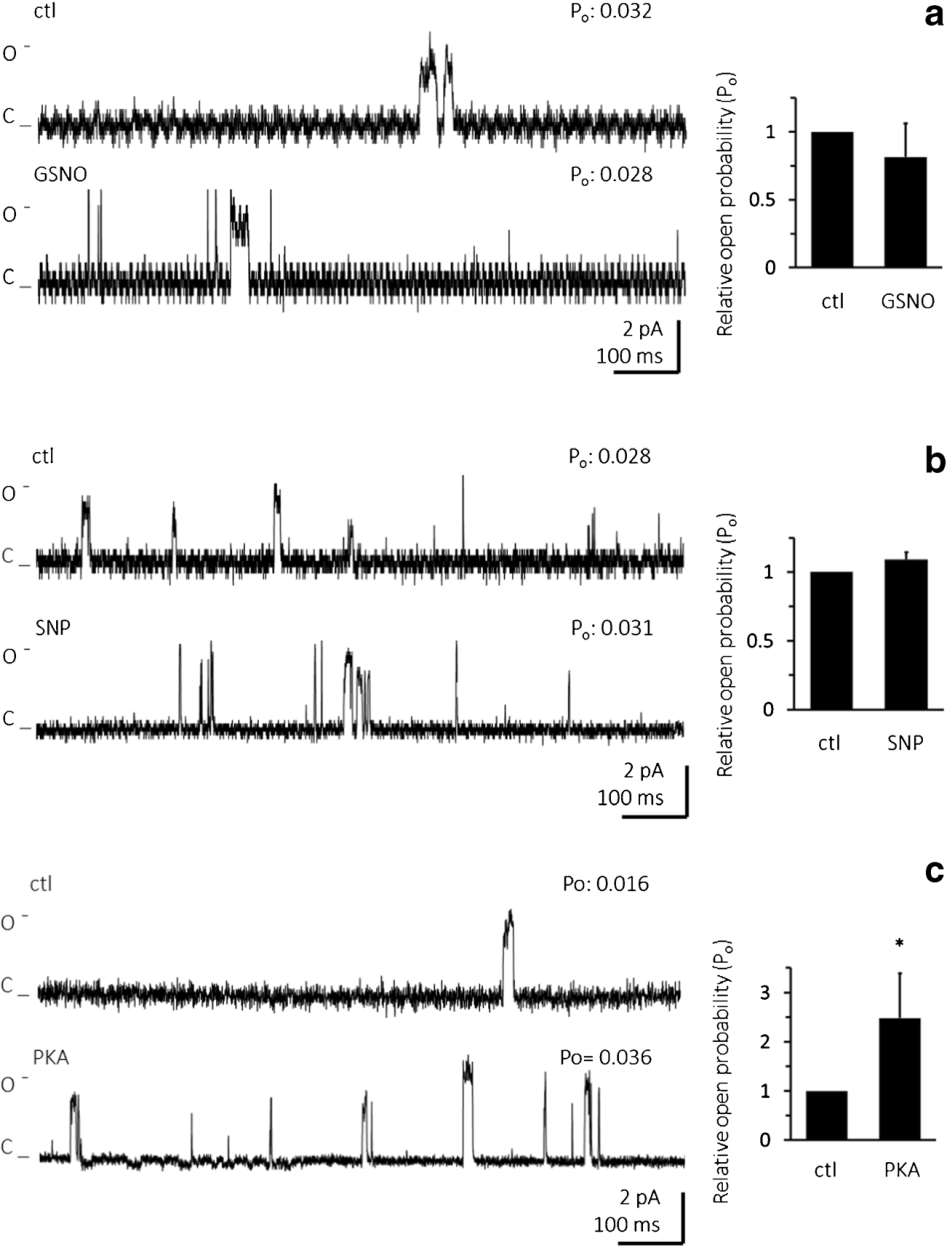
Application of NO donors does not affect single-channel open probability of the long N-terminal isoform of human Ca_v_1.2 channel. **a** Left: representative single-channel currents recorded at + 150 mV in the absence (ctl) or presence of 150 μM NO donor GSNO. Right: relative mean ± SD channel open probability for currents recorded in control solution (ctl) and in the presence of GSNO (*n* = 4, number of liposome patches).**b** NO donor SNP had no effect on single-channel current of the long N-terminal isoform of Ca_v_1.2. Left: representative single-channel currents recorded at + 150 mV in control conditions (ctl), and in the presence of 100 μM SNP. Right: relative mean ± SD channel open probability for currents recorded in control condition, in the presence of SNP (*n* = 6). **c** PKA increases open probability of the single-channel current of the human long N-terminal isoform of Ca_v_1.2. Left: representative single-channel currents recorded at + 150 mV in the absence (ctl) or presence of 0.5 μM PKA (modified after [9]). Right: relative mean ± SD channel open probability for currents recorded in control solution and during PKA treatment (*n* = 7). Single asterisk indicates a significant difference between the control condition and the presence of PKA (*p* < 0.05, paired Student’s *t* test)

In a final set of experiments, we tested the effects of NO donors on single-channel currents through the pore forming α1 subunit of the human Ca_v_1.2 channel incorporated in artificial liposomes. By using this methodological approach, we were able to study potential direct effects of NO on channel function, which, in contrast to our studies on cardiomyocytes, were certainly independent of issues such as sufficient drug diffusion through the myocyte membrane, or the presence of functional signalling pathways in the cells. Figure 6 shows that the presence of two different NO donors (A, GSNO and B, SNP) did not affect Ca_v_1.2 channel open probability. Application of PKA as positive control (Fig. 6c), on the other hand, significantly increased open probability. These results suggest that NO does not exert direct modulatory effects on human Ca_v_1.2 channels.

## Discussion

### nNOS/NO regulation of Ca^2+^ cycling in ventricular cardiomyocytes is independent of Ca_v_1.2 channel modulation

Among the different existing NOS isoforms, nNOS and endothelial NOS (eNOS) are constitutively expressed in cardiomyocytes, whereas expression of inducible NOS (iNOS) only occurs in the presence of injury and inflammation [26]. Recent consensus is that nNOS is the isoform in cardiomyocytes that plays the predominant role in modulating Ca^2+^ cycling and contractility [14, 31, 33]. The commonest view is that NO, generated by nNOS activity, reduces the currents through L-type Ca^2+^ channels [5, 25, 26, 33], which leads to a diminished Ca^2+^ release from the SR, and finally a reduced contractility [5, 19, 25]. This theory—consistent with a positive correlation between Ca^2+^ influx and Ca^2+^ release in cardiomyocytes [3, 8, 10]—however, is not consistent with the findings of several other authors (e.g. [1, 2, 34]; see Introduction).

In the present study, we have carefully reinvestigated potential modulatory effects of nNOS/NO activity on Ca^2+^ cycling and L-type Ca^2+^ channels in ventricular cardiomyocytes. Whereas we were able to confirm the well-described inhibitory effect of nNOS/NO on intracellular Ca^2+^ transients [5, 19, 25], we failed to detect any substantial modulatory effect on basal L-type Ca^2+^ channel activity. Strikingly, in cells originating from the same cardiomyocyte preparations, superfusion with nNOS inhibitors significantly increased the amplitudes of cytosolic Ca^2+^ transients, but did not substantially affect the currents through sarcolemmal Ca^2+^ channels. In addition, Ca_v_1.2 single-channel open probability was unaffected by the presence of NO donors. We therefore propose that, in single isolated ventricular cardiomyocytes, the regulation of Ca^2+^ cycling by nNOS/NO can occur independent of Ca_v_1.2 channel modulation. This further implies that nNOS/NO regulates Ca^2+^ cycling by at least one other mechanism than Ca_v_1.2 channel inhibition. Among the various described NOS regulatory mechanisms of cardiac EC coupling (reviewed in [26, 33]), modulation of ryanodine receptor function in the SR membrane and regulation of SR Ca^2+^ load are obvious candidates [17].

### Potential reasons for inconsistent results in the literature

In contrast to the present study, several authors have demonstrated significant inhibitory effects of nNOS/NO activity on cardiac L-type Ca^2+^ channels (see the “Introduction” section). On the other hand, there is also evidence from other groups against this concept in line with the work presented herein (e.g. [1, 2, 34]). Although we cannot fully explain the apparent inconsistencies, subsequently potential contributing factors are discussed.

Differences in the methodological approaches used in studies may have contributed to inconsistencies in experimental findings. These may have emerged from differences in animal species, age, and gender, as well as varieties in myocyte isolation procedures, applied patch clamp configurations, pulse protocols, experimental solutions, experimental temperatures or drug application approaches (acute application or pre-incubation). Further, we cannot rule out that small inhibitory effects of nNOS/NO activity on Ca_v_1.2 channels exist, which were below the detection threshold of our methodological approaches, but might have been detected in other laboratories. We have tried to find potential correlations between specifically applied methodological approaches and the gained results. This, however, miserably failed due to incomplete descriptions of the experimental procedures in several of the articles cited herein. One noticeable difference between our study and several papers which, in contrast to us, reported increased Ca^2+^ current amplitudes following pharmacological inhibition of nNOS activity in rodent ventricular cardiomyocytes [5, 12, 25, 32] was the absence of Na^+^ in our bath solution. We performed control experiments with a bath solution containing 140 mM Na^+^ (comparable concentration as used in the named studies), which ruled out that this had actually caused the discrepancy in the results. Thus, irrespective of the absence or presence of external Na^+^, in our hands, 100 μM L-VNIO failed to increase the Ca^2+^ currents in mouse ventricular cardiomyocytes (data not shown).

Another factor that may add to the inconsistencies in experimental results in the literature is the actual “β-adrenergic tone” of a cardiomyocyte. Rozmaritsa et al. [23] reported that the effects of the NO donor SNAP on the Ca^2+^ currents in human atrial myocytes are dependent on the intracellular cAMP levels. Similarly, in rat ventricular myocytes, NOS inhibition did not affect the basal Ca^2+^ current, but in a cAMP-stimulated condition, the current was significantly augmented by the application of a NOS inhibitor [18]. In contrast to this study, application of the nNOS inhibitor L-VNIO in our experimental system did not enhance the Ca^2+^ currents in mouse ventricular cardiomyocytes in a cAMP-stimulated condition (Suppl. Fig. 1). Thus, whereas both Matsumoto and colleagues [18] and our present work suggest that NOS/nNOS activity does not regulate the basal activity of L-type Ca^2+^ channels, the situation in case of β-adrenergic stimulation remains controversial. It is possible that a certain “threshold cAMP level” in cardiomyocytes exists, which only when exceeded, allows NOS activity to inhibit Ca^2+^ currents. Since neither Matsumoto and colleagues [18] nor we have measured cellular cAMP levels, it is impossible to verify this hypothesis here. Interestingly, some authors (e.g. [5, 25]) have provided evidence for Ca^2+^ channel inhibition by nNOS activity in cardiomyocytes also in experiments lacking efforts to induce β-adrenergic stimulation. Perhaps, this can occur if isolated myocytes have sufficiently high basal cAMP concentrations. Speculating that nNOS activity inhibits Ca^2+^ currents in a sufficiently cAMP-stimulated condition, our present results suggest that this does occur indirectly, i.e. via signalling cascades, and not via a direct modulatory effect of NO on the Ca_v_1.2 channel. Consistent with this argument, we report here that the presence of NO donors did not affect human Ca_v_1.2 single-channel open probability in artificial liposomes.

In summary, we cannot unequivocally explain the apparent inconsistencies in experimental results in the literature. Owing to our own findings, we propose that basal Ca_v_1.2 channel activity in ventricular cardiomyocytes is not substantially regulated by nNOS/NO. This interpretation is solidly based on the use of different methodological approaches performed in two independent labs (whole cell patch clamp, perforated patch studies (K. Hilber lab); single channel recordings in liposomes (L.C. Hool lab)). Further, these methods were applied on cardiomyocytes and Ca_v_1.2 channels originating from three different species (mouse, rat and human).

### S-Nitrosylation is not a mechanism for significant Ca_v_1.2 post-translational regulation

S-Nitrosylation of cardiac L-type Ca^2+^ channels was suggested to represent a mechanism for Ca^2+^ current inhibition by NOS-derived NO [5, 21, 28, 29]. To the best of our knowledge, however, direct evidence for this hypothesis is lacking. Here, we show that the presence of two different NO donors, expected to induce channel S-nitrosylation, did not affect the human Ca_v_1.2 single-channel open probability (Fig. 6). This suggests that S-nitrosylation of Ca_v_1.2 channels does not lead to channel inhibition, which is further supported by our cardiomyocyte experiments. Thus, neither the NO donor SNAP nor the applied NOS inhibitors exerted any substantial effects on the currents trough Ca^2+^ channels in these cells. Our results are in line with a recent study [34], which has investigated the molecular basis of the regulation of high voltage-activated Ca^2+^ channels, heterologously expressed in HEK cells, by S-nitrosylation. The authors found that, in contrast to currents through Ca_v_2.2 (N-type) Ca^2+^ channels, Ca_v_1.2 currents were hardly affected by the application of the NO donor SNAP. This was attributed to the fact that consensus motifs of S-nitrosylation were much more abundant on Ca_v_2.2 compared with Ca_v_1.2 channels. This study, together with our findings, suggests that S-nitrosylation is not a mechanism for significant Ca_v_1.2 post-translational regulation.

We conclude from our results that—against the currently prevailing view—basal Ca_v_1.2 channel activity in ventricular cardiomyocytes is not substantially regulated by nNOS activity and NO. Hence, nNOS/NO regulation of Ca^2+^ cycling and contractility in myocytes can occur independent of Ca_v_1.2 channel modulation.

## Electronic supplementary material

ESM 1 (PDF 182 kb) External application of the nNOS inhibitor L-VNIO does not affect Ca^2+^ current peaks in mouse ventricular cardiomyocytes in a cAMP-stimulated condition. Top: The rundown-corrected peaks of the currents, elicited by pulses every 3 s, before drug application, during superfusion with bath solution containing 5 μM forskolin and 10 μM milrinone, and finally additionally 100 μM L-VNIO, were plotted over time. Bottom: Evaluation summary of a series of experiments (n = 6) as displayed on top. Ca^2+^ current amplitudes in the absence (ctl) and presence of forskolin and milrinone (Fors + Mil), and additionally L-VNIO (Fors + Mil + L-VNIO) are compared. The control (ctl) amplitude values were determined immediately before forskolin and milrinone application, and the Fors + Mil values were taken just before additional L-VNIO application. The Fors + Mil + L-VNIO amplitude values were always taken 180 s after begin of superfusion with L-VNIO containing bath solution. The current amplitudes are expressed in % relative to the respective value at experiment start (= 100%). Each data point represents a single cell, and data variation is expressed as SD. Cells originated from two mouse hearts.* indicates a significant difference (p < 0.05, paired Student’s t-test) between the drug-free control condition and the presence of forskolin and milrinone. ns, not significant (p = 0.39).
