# Supplementary figures and images for: Neuronal nitric oxide synthase regulation of calcium cycling in ventricular cardiomyocytes is independent of Cav1.2 channel modulation under basal conditions
Source: Pflugers Arch. 2019 Dec 10;472(1):61–74. doi: 10.1007/s00424-019-02335-7 (PMC6960210; doi:10.1007/s00424-019-02335-7)

Suppl. Fig. 1

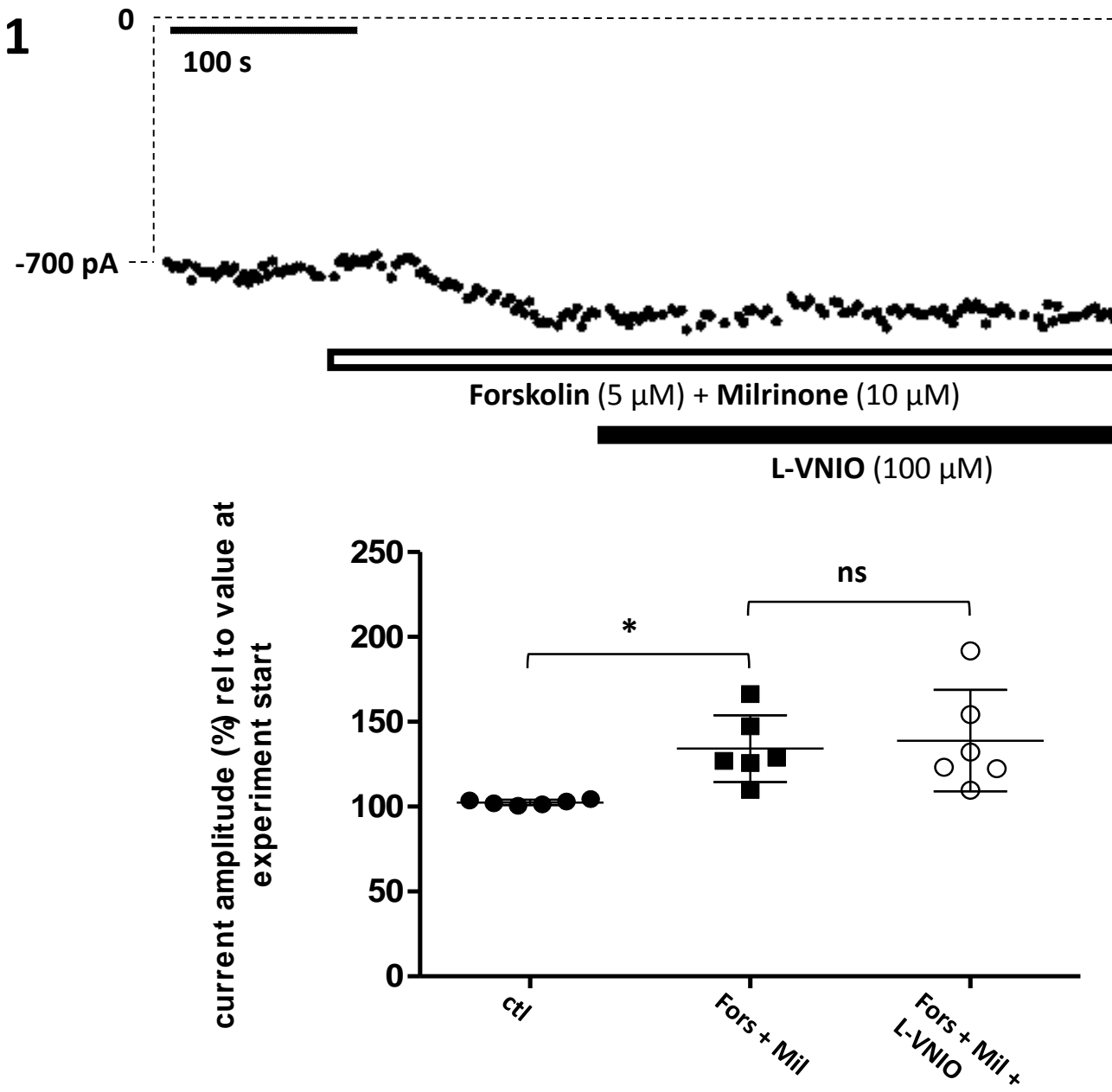

Supplement: Supplementary file 1 — (PDF 182 kb) External application of the nNOS inhibitor L-VNIO does not affect Ca2+ current peaks in mouse ventricular cardiomyocytes in a cAMP-stimulated condition. Top: The rundown-corrected peaks of the currents, elicited by pulses every 3 s, before drug application, during superfusion with bath solution containing 5 μM forskolin and 10 μM milrinone, and finally additionally 100 μM L-VNIO, were plotted over time. Bottom: Evaluation summary of a series of experiments (n = 6) as displayed on top. Ca2+ current amplitudes in the absence (ctl) and presence of forskolin and milrinone (Fors + Mil), and additionally L-VNIO (Fors + Mil + L-VNIO) are compared. The control (ctl) amplitude values were determined immediately before forskolin and milrinone application, and the Fors + Mil values were taken just before additional L-VNIO application. The Fors + Mil + L-VNIO amplitude values were always taken 180 s after begin of superfusion with L-VNIO containing bath solution. The current amplitudes are expressed in % relative to the respective value at experiment start (= 100%). Each data point represents a single cell, and data variation is expressed as SD. Cells originated from two mouse hearts.* indicates a significant difference (p < 0.05, paired Student’s t-test) between the drug-free control condition and the presence of forskolin and milrinone. ns, not significant (p = 0.39). [file 424_2019_2335_MOESM1_ESM.pdf]
